# Correlation between antimicrobial susceptibility data and clinical efficacy for *Helicobacter cinaedi* bacteraemia

**DOI:** 10.1093/jacamr/dlag056

**Published:** 2026-04-15

**Authors:** Hideki Araoka, Junko Tomida, Chiemi Yoshino, Masaru Baba, Sho Ogura, Muneyoshi Kimura, Yoshiaki Kawamura

**Affiliations:** Department of Infectious Diseases, Toranomon Hospital, Tokyo, Japan; Okinaka Memorial Institute for Medical Research, Tokyo, Japan; Department of Microbiology, School of Pharmacy, Aichi Gakuin University, Aichi, Japan; Department of Infectious Diseases, Toranomon Hospital, Tokyo, Japan; Department of Infectious Diseases, Toranomon Hospital, Tokyo, Japan; Department of Infectious Diseases, Toranomon Hospital, Tokyo, Japan; Department of Infectious Diseases, Toranomon Hospital, Tokyo, Japan; Department of Microbiology, School of Pharmacy, Aichi Gakuin University, Aichi, Japan

## Abstract

**Background:**

*Helicobacter cinaedi* is the most common species of enterohepatic *Helicobacter* reported to cause bloodstream infections in humans. The establishment of effective treatment strategies for *H. cinaedi* bacteraemia is required, since the association between *in vitro* susceptibility and clinical response has yet to be demonstrated.

**Methods:**

The medical records of all patients diagnosed with *H. cinaedi* bacteraemia at two Japanese hospitals between March 2009 and December 2016 were retrospectively reviewed. We investigated the antimicrobial susceptibility of *H. cinaedi* using the broth microdilution method and assessed the clinical efficacy of treatment regimens for *H. cinaedi* bacteraemia.

**Results:**

We evaluated the data of 131 patients with first-episode *H. cinaedi* bacteraemia for which MIC data were available for the causative strains. Carbapenems (imipenem and meropenem), aminoglycosides (gentamicin and kanamycin), tetracycline and chloramphenicol exhibited lower MIC values. Fluoroquinolones, especially ciprofloxacin and levofloxacin, demonstrated higher MIC values. Penicillins and cephalosporins showed a wide range of MIC values, from low to high. The MIC_50_ and MIC_90_ of amoxicillin were 4 mg/L and 8 mg/L, respectively. Treatment was successful in 42 of 45 patients (93.3%) who received intravenous β-lactam followed by oral amoxicillin switching therapy or amoxicillin monotherapy. Treatment failed in four of eight patients (50%) in the fluoroquinolone monotherapy group.

**Conclusions:**

Based on our MIC and clinical data, intravenous β-lactam-to-oral amoxicillin switching therapy or amoxicillin monotherapy may be a recommended option, whereas levofloxacin therapy is suboptimal and should be avoided.

## Introduction

*Helicobacter cinaedi* is the most common species of enterohepatic *Helicobacter* reported to cause bloodstream infections in humans.^1,2^ In our previous study summarizing 168 cases of *H. cinaedi* bacteraemia, the overall 30-day mortality rate was low (6.0%), but the 100-day cumulative incidence of recurrent bacteraemia was high (18.7%).^2^ Selective digestive decontamination (SDD) with oral kanamycin was a potential strategy for reducing recurrence^1,3^; however, the establishment of a systemic antimicrobial treatment strategy is expected. The assessment of antimicrobial susceptibility of *H. cinaedi* by the agar dilution^4,5^ and broth microdilution^6^ methods is challenging, and comprehensive data on MIC values remain limited.

The aim of this study was to investigate the antimicrobial susceptibility of *H. cinaedi* using the broth microdilution method, assess the clinical efficacy of treatments for *H. cinaedi* bacteraemia and explore appropriate antimicrobial treatment strategies.

## Materials and methods

### Selection of patients and strains

The medical records of all patients diagnosed with *H. cinaedi* bacteraemia at Toranomon Hospital (868 beds; Tokyo, Japan) and Toranomon Hospital Kajigaya (300 beds; Kanagawa, Japan) between March 2009 and December 2016 were retrospectively reviewed. The culture methods and identification methods for *H. cinaedi* strains have been described previously.^1,2^

### Measurement of antimicrobial susceptibility data

A broth microdilution method using modified Levinthal broth in a 96-well microtiter plate format was performed as previously described.^6^ MICs were determined for the following 19 antimicrobial agents: ampicillin, ampicillin/sulbactam, amoxicillin, carbenicillin, piperacillin, piperacillin/tazobactam, ceftriaxone, cefepime, imipenem, meropenem, gentamicin, kanamycin, tetracycline, erythromycin, ciprofloxacin, levofloxacin, moxifloxacin, metronidazole, and chloramphenicol. Ampicillin and sulbactam were combined in a 2:1 ratio, and the MIC of ampicillin was recorded. In the combination of piperacillin and tazobactam, tazobactam was fixed at 4 mg/L, and the MIC of piperacillin was recorded.

### Clinical efficacy

The clinical evaluation focused on cases of first-episode bacteraemia in which the MICs of the causative strain cultured from blood samples were determined. Treatment failure was defined as either death within 30 days from the onset of bacteraemia or the recurrence of bacteraemia. Recurrent bacteraemia was deﬁned as a positive *H. cinaedi* blood culture of a specimen collected ≥2 days after clinical improvement and/or completion of a course of systemic antimicrobial therapy for *H. cinaedi*.^1^ The administered antimicrobial agents were investigated, and drugs administered as definitive therapy were defined as therapeutic antimicrobial agents. In addition, oral kanamycin as SDD was also investigated.^1^ Statistical analyses were performed using EZR (Saitama Medical Center, Jichi Medical University), which is a graphical user interface for R (R Foundation for Statistical Computing, Vienna, Austria).^7^

This study was approved by the human ethics review committees of Toranomon Hospital, Toranomon Hospital Kajigaya and Aichi Gakuin University.

## Results

### Selection of patients and strains

We identified 168 patients with *H. cinaedi* bacteraemia during the study period. The surviving strains among the stored *H. cinaedi* strains were evaluated. A total of 131 *H. cinaedi* strains from patients with first-episode bacteraemia were analysed.

### Measurement of antimicrobial susceptibility data

The distribution of MICs and MIC_50_/MIC_90_ for all 131 *H. cinaedi* isolates are presented in Table 1. Carbapenems, aminoglycosides, tetracyclines and chloramphenicol exhibited lower MICs, and the MIC_50_/MIC_90_ values of imipenem, meropenem, gentamicin, kanamycin, tetracycline and chloramphenicol were 0.06/0.12 mg/L, 0.06/0.25 mg/L, 0.25/0.5 mg/L, 0.5/1 mg/L, 0.06/0.5 mg/L and 0.25/0.5 mg/L, respectively. Penicillins, cephalosporins and metronidazole showed a wide range of MICs, from lower to higher. The MIC_50_/MIC_90_ values of ampicillin, ampicillin/sulbactam, amoxicillin, carbenicillin, piperacillin, piperacillin/tazobactam, ceftriaxone, cefepime and metronidazole were 16/64 mg/L, 16/32 mg/L, 4/8 mg/L, 16/64 mg/L, 4/8 mg/L, 4/8 mg/L, 4/8 mg/L, 8/16 mg/L and 2/>64 mg/L, respectively. Macrolides, and fluoroquinolones, especially ciprofloxacin and levofloxacin, demonstrated higher MICs. The MIC_50_/MIC_90_ values of erythromycin, ciprofloxacin, levofloxacin and moxifloxacin were 64/>64 mg/L, 64/>64 mg/L, 8/>64 mg/L and 1/16 mg/L, respectively.

**Table 1.** Distribution of MIC values and MIC_50_/MIC_90_ for the 131 *H. cinaedi* isolates from blood samples in patients with first-episode *H. cinaedi* bacteraemia

Antimicrobial agents	MIC values (mg/L)												MIC_50_ (mg/L)	MIC_90_ (mg/L)
	0.06	0.12	0.25	0.5	1	2	4	8	16	32	64	>64		
Ampicillin					1	3	7	23	45	30	21	1	16	64
Ampicillin/sulbactam^a^					1	2	6	22	36	26	10		16	32
Amoxicillin				5	13	14	64	31	3		1		4	8
Carbenicillin	1			1	1	3	10	28	40	32	14	1	16	64
Piperacillin			1	6	8	19	33	54	8	1		1	4	8
Piperacillin/tazobactam			1	6	11	18	49	38	6	1		1	4	8
Ceftriaxone	1		3	6	11	20	52	29	7	1	1		4	8
Cefepime					4	10	24	55	28	6	4		8	16
Imipenem	115	10	4	1	1								0.06	0.12
Meropenem	81	32	14	2	1	1							0.06	0.25
Gentamicin	32	13	49	26	7	3						1	0.25	0.5
Kanamycin	19	11	15	47	27	9	2					1	0.5	1
Tetracycline	69	22	22	13	1	2	1	1					0.06	0.5
Erythromycin								4	16	39	16	56	64	>64
Ciprofloxacin							1	3	12	35	60	20	64	>64
Levofloxacin				1	4	12	18	33	4	4	33	22	8	>64
Moxifloxacin	16	8	5	21	21	4	7	20	19	9	1		1	16
Metronidazole	29	5	4	14	11	5	2	1	5	7	28	20	2	>64
Chloramphenicol	37	20	33	32	7	1	1						0.25	0.5

MIC_50_; 50% minimal inhibitory concentration, MIC_90_; 90% minimal inhibitory concentration.

^a^Ampicillin/sulbactam: 103 isolates.

### Clinical efficacy

Of the 131 cases, 100 were classified as treatment successes, 24 experienced recurrent bacteraemia and seven patients died within 30 days; therefore, it was considered that treatment failure occurred in 31 cases (23.7%). Therapeutic antimicrobial agents used are presented in Table S1 (available as Supplementary data at *JAC-AMR* Online). Of the 131 cases, 34 cases received intravenous β-lactam followed by oral amoxicillin switching therapy, and 11 patients were treated with amoxicillin monotherapy. Intravenous β-lactam monotherapy and fluoroquinolone monotherapy were administered in 25 cases and eight cases, respectively. Thirty-five patients were treated with SDD to prevent recurrence.

### Intravenous β-lactam-to-oral amoxicillin switching therapy or amoxicillin monotherapy

In the intravenous β-lactam-to-oral amoxicillin switching therapy group (*n* = 34), the median duration of amoxicillin administration was 16 days (range: 7–49 days), whereas in the amoxicillin monotherapy group (*n* = 11), the median duration was 21 days (range: 8–35 days). In the intravenous β-lactam-to-oral amoxicillin switching therapy group, the median durations of ampicillin/sulbactam, cefepime, piperacillin/tazobactam and meropenem administration were 11.5 days (range: 4–22 days), 6.5 days (range: 3–15 days), 5.5 days (range: 3–10 days) and 11 days (range: 10–21 days), respectively.

**Figure 1.**
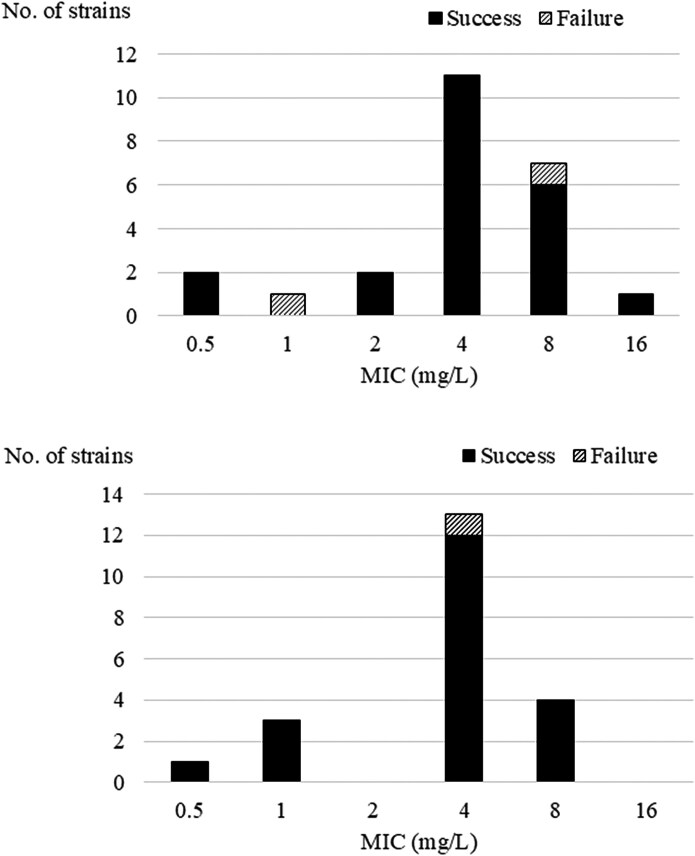
(a) MIC values of amoxicillin and treatment outcomes in 24 cases without SDD treatment. SDD, selective digestive decontamination. (b) MIC values of amoxicillin and treatment outcomes in 21 cases with additional SDD treatment. SDD, selective digestive decontamination.

Figure 1a shows MICs of amoxicillin and outcomes in 24 non-SDD cases, with a 91.7% success rate. Figure 1b presents corresponding data for 21 SDD cases, with a 95.2% success rate. Overall, treatment was successful in 42 of 45 cases (93.3%); all three cases of treatment failure were due to recurrent bacteraemia. The treatment success rate in this group (93.3%) was significantly higher than that in the other groups (67.4%; 58/86) (odds ratio, 6.68; 95% confidence interval. 1.87–36.6; *P* < 0.001)

### Intravenous β-lactam monotherapy

Table S2 summarizes the MICs of β-lactam agents and the treatment outcomes of monotherapy with each β-lactam agent. Although a higher treatment success rate was observed in the groups receiving cefepime or meropenem, this trend did not achieve statistical significance.

### Fluoroquinolone monotherapy

All patients in the fluoroquinolone monotherapy group received levofloxacin. Table S3 summarizes the MICs of levofloxacin and the treatment outcomes of eight patients who received levofloxacin therapy. Treatment failure occurred in four of the eight cases (50%).

## Discussion

To our knowledge, this represents the largest-scale investigation to date linking MICs with treatment outcomes in this setting. Our new findings demonstrate the efficacy of oral amoxicillin-containing regimens for treating *H. cinaedi* bacteraemia in a real-world setting.

The antimicrobial susceptibility results were largely consistent with findings from previous reports,^4,8^ which showed lower MICs for carbapenems, aminoglycosides, tetracyclines and chloramphenicol. Kanamycin demonstrated lower MICs in all but two strains, suggesting that SDD with oral kanamycin may be a reasonable treatment option for preventing recurrence. However, it was not possible to evaluate the impact of kanamycin resistance on the clinical response in this study. Ampicillin, ampicillin/sulbactam, amoxicillin, carbenicillin, piperacillin, piperacillin/tazobactam, ceftriaxone and cefepime showed a wide range of MICs, from lower to higher. The MIC_50_ and MIC_90_ values of amoxicillin were 4 mg/L and 8 mg/L, respectively. The MIC_90_ values of amoxicillin were reported to range from 4 to 8 mg/L in earlier studies^8,9^; otherwise, the sample sizes were small. Although no official breakpoints have been established for *H. cinaedi*, the CLSI and EUCAST define the susceptibility breakpoint for amoxicillin (as amoxicillin/clavulanic acid in CLSI) against Enterobacterales as  ≤ 8 mg/L.^10,11^ Based on these reference MICs, amoxicillin is expected to demonstrate clinical efficacy. Previous studies reported lower MICs for amoxicillin and amoxicillin/clavulanate than for ampicillin, consistent with our findings.^4,8^ The comparable MICs between ampicillin and ampicillin/sulbactam, as well as between piperacillin and piperacillin/tazobactam, suggest a low likelihood of β-lactamase production. Fluoroquinolones, particularly ciprofloxacin and levofloxacin, and macrolides exhibited higher MICs, consistent with previous reports.^8^

When comparing *in vitro* data with clinical outcomes, two notable trends were observed: first, levofloxacin exhibited higher MICs and was associated with a higher rate of treatment failure; second, amoxicillin showed MICs ≤ 8 mg/L in most isolates, with a low rate of treatment failure regardless of whether SDD was performed. One possible explanation for the low rate of treatment failure in patients receiving oral amoxicillin is the relatively long period for which it was administered. Some previous studies have recommended long-term over short-term therapy; accordingly, a treatment duration of  ≥ 21 days may be appropriate.^12–14^

This study had some limitations. First, it was a retrospective study. Not all potential confounding factors could be controlled for. A prospective study utilizing a single antimicrobial agent with a standardized treatment duration is warranted. Nevertheless, conducting prospective studies on this rare disease is challenging, and the existing *in vitro* and clinical efficacy data are considered especially valuable. Second, potential issues exist regarding the definition of treatment failure. General guidelines for the clinical evaluation of anti-infective drug products reported that outcomes should be determined by predefined clinical, microbiological, laboratory, radiological or other pertinent endpoints.^15^ This pathogen typically requires a median of 5 days to grow in blood culture, and its clinical presentation is often limited to fever alone.^2^ As a result, assessing the resolution of clinical symptoms was challenging. Also, blood cultures were performed only in cases of recurrent infection, with most patients presenting with fever. Accordingly, the true incidence of recurrent bacteraemia may be substantially underestimated.

### Conclusions

Based on our MIC and clinical data, intravenous β-lactam-to-oral amoxicillin switching therapy or amoxicillin monotherapy may be considered as a recommended option, whereas levofloxacin therapy is suboptimal and should be avoided.

## Supplementary Material

dlag056_Supplementary_Data

## Supplementary data

Tables S1–S3 are available as Supplementary data at *JAC-AMR* Online.
