# Supplementary material for: Correlation between antimicrobial susceptibility data and clinical efficacy for Helicobacter cinaedi bacteraemia
Source: JAC Antimicrob Resist. 2026 Apr 15;8(2):dlag056. doi: 10.1093/jacamr/dlag056 (PMC13079442; doi:10.1093/jacamr/dlag056)
Supplement: dlag056_Supplementary_Data [file dlag056_supplementary_data.docx]

**Supplementary material**

**Table S1. Therapeutic antimicrobial regimens used for the 131 first episodes of bacteraemia**

| Therapeutic antimicrobial agents | No. (n = 131) |
| --- | --- |
| Amoxicillin containing therapy | 45 |
| Intravenous β-lactam-to-oral amoxicillin switching therapy | 34 |
| Amoxicillin monotherapy | 11 |
| Intravenous β-lactam monotherapy | 25 |
| Ampicillin/sulbactam | 5 |
| Piperacillin/tazobactam | 4 |
| Ceftriaxone | 8 |
| Cefepime | 4 |
| Meropenem | 4 |
| Fluoroquinolone monotherapy | 8 |
| Levofloxacin | 8 |
| Others (e.g. antimicrobial agents without corresponding MIC data,  multiple antimicrobial agents including β-lactam and/or fluoroquinolone) | 50 |
| None | 3 |

**Table S2. Clinical efficacy of intravenous β-lactam monotherapy**

| MICs of  ampicillin/sulbactam (mg/L) | 8 | 8 | 8+SDD | 16 | 64+SDD |  |  |  |
| --- | --- | --- | --- | --- | --- | --- | --- | --- |
| Outcome | Failure | Failure | Success | Success | Success |  |  |  |
| MICs of  piperacillin/tazobactam (mg/L) | 4 | 8 | 8 | 8+SDD |  |  |  |  |
| Outcome | Failure | Success | Failure | Success |  |  |  |  |
| MICs of  ceftriaxone (mg/L) | 2+SDD | 4 | 4 | 4 | 4+SDD | 8 | 8+SDD | 64 |
| Outcome | Success | Success | Failure | Failure | Success | Success | Failure | Success |
| MICs of  cefepime (mg/L) | 1 | 2 | 8 | 8 |  |  |  |  |
| Outcome | Success | Success | Success | Success |  |  |  |  |
| MICs of  meropenem (mg/L) | 0.06 | 0.06 | 0.06 | 0.12 |  |  |  |  |
| Outcome | Success | Success | Failure | Success |  |  |  |  |

SDD, selective digestive decontamination

**Table S3. Clinical efficacy of levofloxacin monotherapy**

| MICs of  levofloxacin (mg/L) | 2 | 4+SDD | 4 | 8 | 64 | >64 | >64 | >64 |
| --- | --- | --- | --- | --- | --- | --- | --- | --- |
| Outcome | Success | Success | Failure | Failure | Failure | Success | Success | Failure |

SDD, selective digestive decontamination
